# Identification and genome analysis of tomato chlorotic spot virus and dsRNA viruses from coinfected vegetables in the Dominican Republic by high-throughput sequencing

**DOI:** 10.1186/s12985-018-0931-9

**Published:** 2018-01-26

**Authors:** Reina Teresa Martínez, Mariana Martins Severo de Almeida, Rosalba Rodriguez, Athos Silva de Oliveira, Fernando Lucas Melo, Renato Oliveira Resende

**Affiliations:** 1 Universidad Autónoma de Santo Domingo-UASD and Instituto Dominicano de Investigaciones Agropecuarias y Florestales – IDIAF, Santo Domingo, Dominican Republic; 2 Departamento de Biologia Celular, Universidade de Brasília, Brasilia, Brazil; 3 Universidad Autónoma de Santo Domingo-UASD, Santo Domingo, Dominican Republic

**Keywords:** Dominican Republic, Tospovirus, dsRNA viruses, Vegetables, Mixed infection

## Abstract

The Tomato chlorotic spot virus (TCSV) was first reported in the 1980s, having its occurrence limited to Brazil and Argentina. Due to an apparent mild severity in the past, molecular studies concerning TCSV were neglected. However, TCSV has disseminated over the USA and Caribbean countries. In Dominican Republic TCSV has been recently reported on important cultivated crops such as pepper and beans. In this work, we provide the first complete genome of a TCSV isolate from symptomatic plants in Dominican Republic, which was obtained by high-throughput sequencing. In addition, three dsRNA viruses from different virus families were identified coinfecting these plants *Bell pepper endornavirus* (BPEV), *Southern tomato virus* (STV) and *Pepper cryptic virus 2* (PCV-2). Phylogenetic analysis showed that the Dominican Republic TCSV isolate has a close relationship with other TCSV isolates and a reassortant isolate between TCSV and Groundnut ringspot virus (GRSV), all found in USA. BPEV, STV and PCV-2 isolates from Dominican Republic were close related to corresponding American isolates. The possible biological implications of these virus-mixed infections are discussed.

## Background

Dominican Republic (DR) contains large areas of vegetable production, which bases a significant part of this country economy. Chillies and peppers are amongst the top ten vegetable products exported by DR according to the Observatory of Economic Complexity (OEC), a tool that allows to quickly composing a visual narrative about countries and the products they exchange [1]. Recently, this vegetable production has been threatened by tospoviruses infections, viruses that belong to genus *Tospovirus*, family *Bunyaviridae* [2]. While *Tomato chlorotic spot virus* (TCSV) have caused typical tospovirus symptoms in chili pepper (*Capsicum frutescens*), long beans (*Vigna unguiculata*) and tomatoes (*Solanum lycopersicum*), *Tomato spotted wilt virus* (TSWV) and TCSV as well have been found in potatoes (*S. tuberosum*), fresh and processing tomatoes and sweet pepper (*C. annuum*) [3–5]. These tospovirus species are notorious for inducing substantial losses on vegetable production around the world [6].

Although frequent reports of new virus species and virus hosts are available, the real diversity of plant viruses has been overlooked for a long period. Only plants with economic importance and presenting compromising symptoms have mostly been surveyed for identification of disease causative agents. With the accessibility of high-throughput sequencing tools, this scenario has changed and viruses, which causing no apparent disease symptoms, have been found in large scales [7–10]. These findings have confirmed the hypothesis that pathogenic viruses would rather be an exception than a rule. Some viruses have even been proved to turn plants more tolerant to drought, heat and cold [11, 12]. Overall, the biological meaning of these mixed infections still has to be elucidated.

In this study sequences covering virus-derived genomes were retrieved from RNA sequencing data of symptomatic vegetables collected in DR. These samples were those previously shown to be positive for TCSV and TSWV [4]. Here we report the first complete genome of a TCSV isolate from DR, in addition to three genomes of dsRNA viruses coinfecting vegetable crops.

## Material and methods

**Fig. 1:**
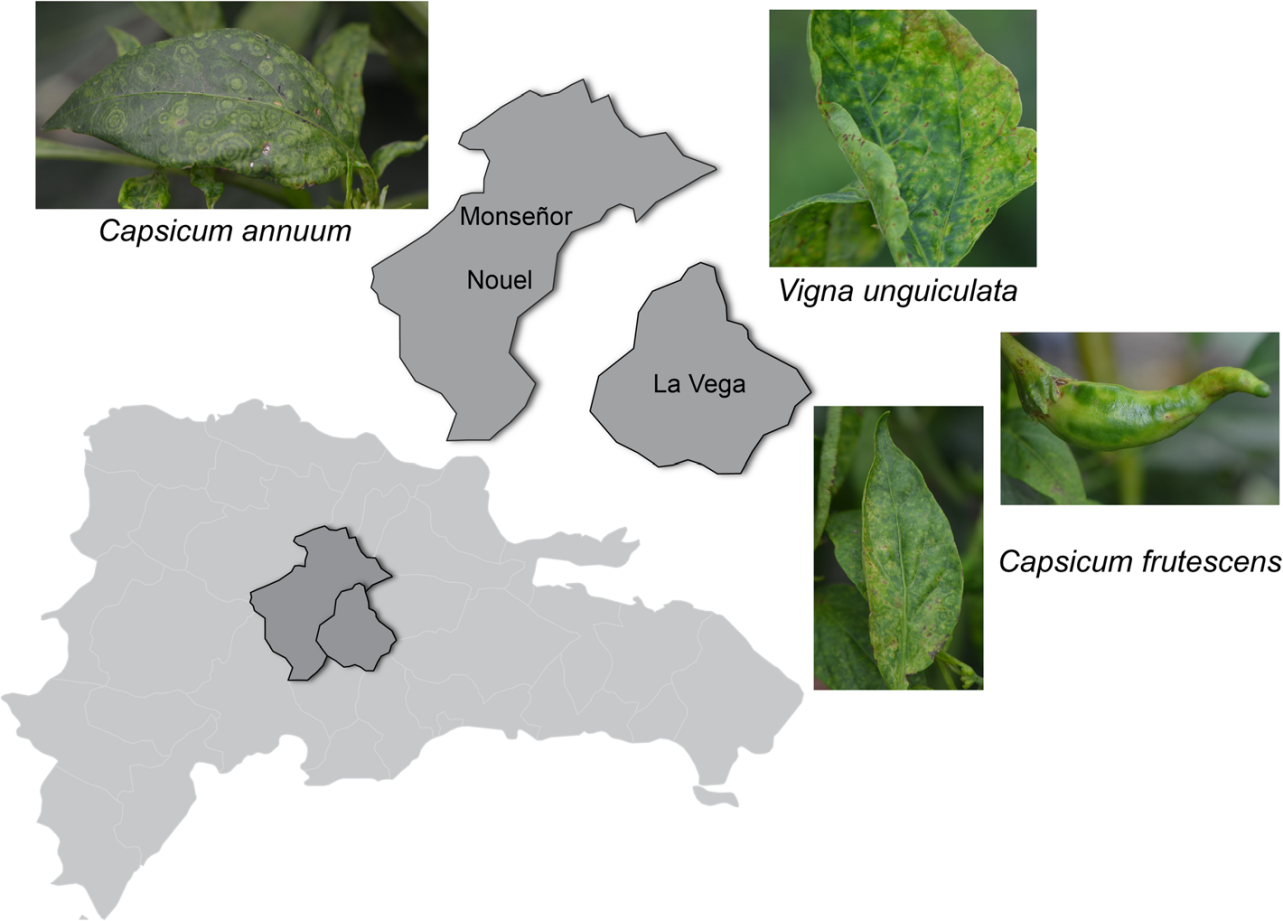
The Provinces in Dominican Republic where symptomatic samples were collected. Sweet pepper (*Capsicum annuum*) collected in Monseñor Nouel province showing the typical tospovirus symptom on leaves, the chlorotic rings. Chilli pepper (*Capsicum frutescens*) from La Vega province showing twisted fruits and chlorotic rings on leaves. Long beans (*Vigna unguiculata*) collected in La Vega province presenting similar chlorotic and necrotic spots on leaves

Leaf samples of symptomatic tomato (*Solanum lycopersicum*), potato (*S. tuberosum*), long beans (*Vigna unguiculata*) and chili pepper (*Capsicum frutescens*) were collected in the province of La Vega, and sweet pepper (*C. annuum*) in the province of Monseñor Nouel (Fig. 1). Total RNA isolation was performed using the commercial kit mirVana™ (Ambion™) to improve RNA quality sent for sequencing. Aliquots of each sample were pooled to compose a single sample.

Whole transcriptome shotgun sequencing of the RNA pool was done using an Illumina Hi Seq 2000 platform, which ended up in the production of about 53 million reads. The paired-ends reads were quality-filtered, the adapter sequences were removed, and contigs were assembled *de novo* using CLC Genomics Workbench version 6.0.3. Contigs covering virus-derived genomes were built by BLASTn and BLASTx searches against the virus reference database available in the National Center for Biotechnology Information (NCBI). The Geneious software was used for further characterization and BLASTx searches.

Specific primers were designed to determine by RT-PCR which plants were infected with the viruses found in the deep sequencing analysis (Table 1). The total RNA extracted from tomato, potato, long bean, chilli pepper and sweet pepper were used as template for cDNA synthesis. For first-strand cDNA synthesis, 2 μl of each RNA were mixed with 1 μl specific forward and reverse primer pair [10 μM], 1 μl dNTPs solution [10 mM] and 8 μl RNAse free water under 70 °C during 5 min, followed by a rapid cooling on ice. Then, it was added per reaction 2 μl [5×] First Strand® Buffer, 1 μl DTT (DL-Dithiothreitol) [0.1 M], 0.5 μl Moloney Murine Leukemia Virus Reverse Transcriptase (M-MLV RT) (Invitrogen) [200 units./μl], and water up to a final volume of 20 μL. The reaction was incubated at 37 °C for 1 h, followed by a final denaturation at 70 °C for 15 min. For PCR, *Taq* Platinum® DNA polymerase (Invitrogen) was used as it follows: 1.5 μl MgCl_2_ [50 Mm], 2.5 μl 10X buffer, 0.5 μl primer forward [10 μM], 0.5 μl primer reverse [10 μM], 0.5 μl dNTPs solution [10 mM], 0.1 μl *Taq* DNA polymerase, 1 μl cDNA, and water up to 25 μL. The amplification program consisted of a primary denaturation at 94 °C for 2 min, followed by 35 cycles at 94 °C for 30 s, 55 °C for 30 s and 72 °C for 1 min, and one step final extension at 72 °C for 5 min. PCR products were sent for Sanger sequencing at Macrogen Inc. (South Korea). The sequences were then compared with those deposited in the GenBank database via BLAST.

**Table 1:** Primers used for amplification of virus sequences of symptomatic vegetables from Dominican Republic

Primer Name	Sequence 5ʼ - 3ʼ	Tm [°C]	Anneling Temperature [°C]
Amalga – F^a^	TGG GTA TCG ACA AGC GCT AC	60.5	55
Amalga – R^a^	ACA TGT CGA AGG CCT CCT TG	60.5	55
Bell – F^b^	CGC TTC GAG CAT AAA AGC CC	60.5	55
Bell – R^b^	TGG CTT GCG CTT TTG TGT AC	58.4	55
Pep70 – F^c^	CAC CCG CAC ACA ATT AAC GG	60.5	55
Pep70 - R^c^	ACA CAT CTT CGG TCC GAC AC	60.5	55
Pep126 – F^d^	ACG CCC CCT ATA ACG CAA AA	58.4	55
Pep126 – R^d^	AAT GTC GCA AGG GCC CAT AA	58.4	55

Melting temperature (Tm) and annealing temperature in PCR reaction are shown

^a^Primers to amplify *Southern tomato virus* (STV)

^b^Primers to amplity *Bell pepper endornavirus* (BPEV)

^c^Primers to amplity *Pepper cryptic virus 2* (PCV-2) RNA 1

^d^Primers to amplify *Pepper cryptic virus 2* (PCV-2) RNA 2

For phylogenetic analysis, multiple alignments were performed by MUSCLE implemented in Seaview v.4.5.4 and the phylogenetic trees were built by PhyML software [13–15]. Maximum Likelihood was used as the criterion to infer phylogenetic relationships between the isolates. The appropriate nucleotide substitution model was selected by JmodelTest program version 2.1.4 [16]. The visualization and edition of the phylogenetic trees were performed using FigTree v.1.4.1.

## Results

**Fig. 2:**
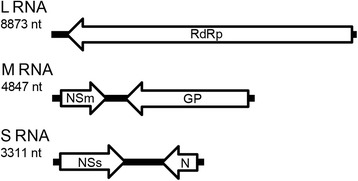
Genome representation of Tomato chlorotic spot virus (TCSV) isolated from Dominican Republic. The L RNA has a negative polarity with 8873 nucleotides that encodes to the viral RNA polymerase (RdRp). The M RNA has 4847 nucleotides and two ORFs in ambisense orientation that encode to the envelope glycoproteins (Gn and Gc) in the virus- complementary sense and the movement protein (Nsm) in the viral strand. The S RNA with 3311 nucleotides is ambisense as well. The viral strand encodes to the non-structural protein (Nss) and the complementary strand the nucleocapsid protein (N). The expected sizes of TCSV-encoded proteins are indicated. The consensus of each genomic segment was deposited under GenBank accession numbers KX463272 [Large (L) segment], KX463273 [Medium (M) segment], and KX463274 [Small (S) segment]

**Fig. 3:**
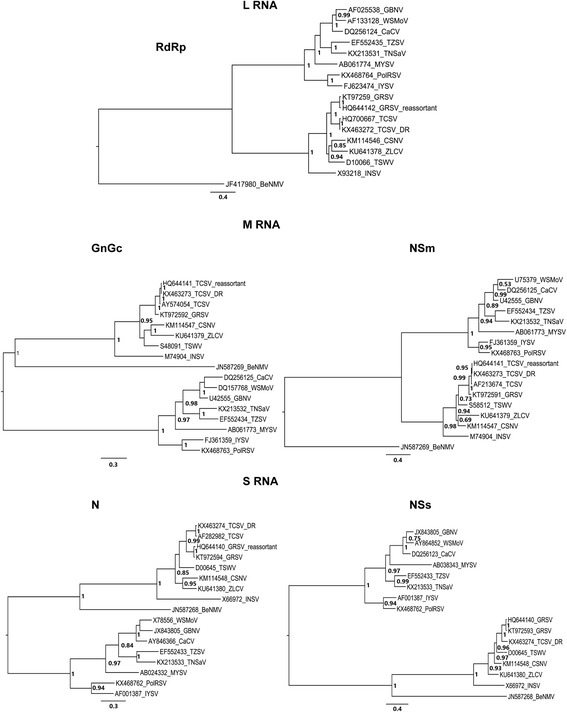
*Tospovirus* phylogeny. Trees were based on amino acid sequences of the structural proteins RNA-dependent RNA polymerase (RdRp), the nucleocapsid (N) and the glycoprotein (GnGc) and the non-structural movement (Nsm) and the silence suppressor (Nss) proteins. The species included in the phylogenetic analysis were Bean necrotic spot virus (BeNMV), Capsicum chlorosis virus (CaCV), *Groundnut bud necrosis virus* (GBNV), *Groundnut ringspot virus* (GRSV), *Groundnut ringspot virus*/*Tomato chlorotic spot virus* (reassortant) (GR/TC), Chrysanthemum stem necrosis virus (CSNV), *Impatiens necrotic spot virus* (INSV), *Iris yellow spot virus* (IYSV), Melon yellow spot virus (MYSV), *Polygonum ringspot virus* (PolRSV), *Tomato chlorotic spot virus* (TCSV), Tomato necrotic spot-associated virus (TNSaV), *Tomato spotted wilt virus* (TSWV), Tomato zonate spot virus (TZSV), *Watermelon silver mottle virus* (WSMoV) and *Zucchini lethal chlorosis virus* (ZLCV). The GenBank accession numbers of the sequences are shown in the trees. Nodes values with posterior probability > 50% are shown. Protein sequences were used to verify the phylogenetic relationship of the *Tomato chlorotic spot virus* (TCSV) isolate reported in Dominican Republic (GenBank accession numbers KX463272 [L RNA], KX463273 [M RNA], KX463274 [S RNA]). The proteins encoded at S and L RNAs clustered with the TCSV isolate already reported (GenBank accession numbers AF282982 [S RNA] and HQ700667 [L RNA]), while those encoded at M RNA are closer to the hybrid North American TCSV/GRSV (reassortant) isolate (GenBank accession number HQ644141)

A complete genome of the tospovirus *Tomato chlorotic spot virus* (TCSV) was assembled (Fig. 2). Tospoviruses are compound by three single stranded-RNAs within spherical particle of 80-120 nm diameter [2]. The consensus of each genomic segment was deposited under GenBank accession numbers KX463272 [Large (L) segment], KX463273 [Medium (M) segment], and KX463274 [Small (S) segment]. The L segment, which has a negative polarity, encodes a RNA-dependent RNA polymerase of 2874 amino acids (aa). The M segment codes for the viral movement protein (NSm) of 303 aa and for the glycoprotein precursor (GP) of 1134 aa in an ambisense genomic organization. As expected, the same ambisense polarity is observed for the S segment, which codes for the nucleocapsid (N) protein of 258 aa and a putative RNA silencing suppression protein (NSs) of 469 aa (Fig. 2). Pairwise comparisons of the N, NSs and RdRp protein sequences showed that TCSV-DR is most closely related to the TCSV isolates reported in cultivated and ornamental plants [17, 18]. The NSm and the glycoprotein precursor protein sequences analysis revealed TCSV-DR closest to the unique reassortant isolate formed between TCSV and GRSV (S_GRSV_, M_TCSV_ and L_GRSV_) (Fig. 3) [19].

The sequencing also revealed RNA genomes of dsRNA viruses from three different genera, apart from the complete genome of TCSV and partial sequences of TSWV. All virus sequences matched with species previously reported infecting crops (Table 2).

**Table 2:** Viruses species sequenced from the RNA pool and traced back in infected vegetables

Virus Species	Virus Family	DR Host^a^	Acession Number	Genome Type
*Bell pepper endornavirus* (BPEV)	*Endornaviridae*	Sweet Pepper	KX525267	dsRNA
*Pepper cryptic virus 2* (PCV-2) (bipartite)	*Partitiviridae*	Chilli Pepper	KX525268 (RNA1)	dsRNA
			KX525269 (RNA 2)	
*Southern tomato virus* (STV)	*Amalgaviridae*	Tomato	KX525266	dsRNA
*Tomato chlorotic spot virus* (TCSV) (tripartite)	*Bunyaviridae*	Chilli Pepper; Long Beans	KX525272 (L RNA)	ssRNA
			KX525273 (M RNA)	
			KX525274 (S RNA)	

^a^DR-Host – Comercial vetables coinfected by tospovirus and other dsRNA viruses in Dominican Republic

**Fig. 4:**
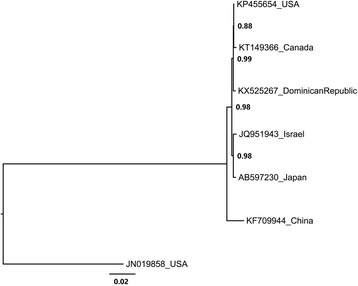
*Bell pepper endornavirus* (BPEV) phylogeny. The tree based on nucleotide sequences of BPEV available on GenBank. Complete nucleotide sequences of BPEV isolates deposited in GenBank were used to analyse the phylogenetic relationship of the isolate found in Dominican Republic (GenBank accession number KX525267). The isolates are represented in the tree by the accessions numbers of the sequences and the collection point. Nodes values with posterior probability > 50% are shown. The Dominican Republic isolate is shown to be more phylogenetic related to others Americans isolates, as the Canadian (GenBank accession number KT149366) and the North American (GenBank accession number KP456554)

A contig corresponding to a complete sequence of a *Bell pepper endornavirus* (BPEV-DR) isolate (Family *Endornaviridae*) was found and traced back in chilli pepper (*C. frutescens*) (GenBank accession no. KX525267). The viruses from family *Endornaviridae* present a dsRNA genome that range in length from about 14kbp to 17.6kbp [20]. The genome of BPEV-DR consists of 14,790 nucleotides in size, coding for a single open reading frame (ORF) starting at nucleotide (nt) 23 and ending at the nt 14,677, which could encode for a polyprotein of 4884 amino acids (aa). The putative BPEV-DR protein product shared 92–99% similarity with the single ORF of BPEV already reported. Multiple alignment with complete endornaviruses sequences avaiable on the GenBank database showed that BPEV-DR presents 87%–99% nucleotide identity with BPEV isolates found in America, Asia and Middle East [7, 10, 21–23, 24]. Phylogenetic analysis using whole genome showed that BPEV-DR clustered in a separate group with the American isolate Maor (GenBank accession no. KP455654) and a Canadian isolate (GenBank accession no. KT149366) (Fig. 4).

**Fig. 5:**
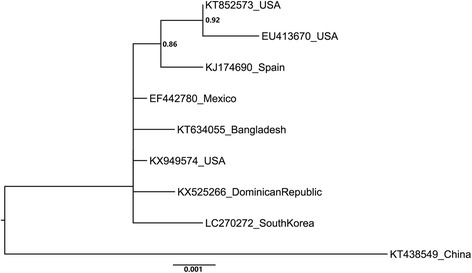
*Southern tomato virus* (STV) phylogeny. The tree was based on nucleotide sequences of STV available on GenBank. Complete nucleotide sequences of STV isolates deposited in GenBank were used to analyse the phylogenetic relationship of the isolate found in Dominican Republic (GenBank accession number KX525266). The isolates are represented in the tree by the accessions numbers of the sequences and the collection point. Nodes values with posterior probability > 50% are shown. Due to the low genetic variability, the exact phylogenetic relationship of the Dominican Republic isolate with other STV isolates could not be define. It establishes relationship with Americans and Asiatic isolates as well

A *Southern tomato virus* (STV-DR) isolate was found infecting tomato (GenBank accession no. KX525266). The STV is classified within the genus *Amalgavirus* (family *Amalgaviridae*), usually presenting a dsRNA genome of about 3.5 kb that encodes two overlapping ORFs [2, 25]. The STV-DR genome has 3.425 kb and shares more than 99% nucleotide identity with other STV isolates from America, Asia and Europe [25, 26]. Analysis of the STV-DR genome showed the presence of the two overlapping ORFs. The ORF 1 encodes a 377 aa putative coat protein, initiated at nt 126 and ending at nt 1257. Another ORF encoding a protein of 762 aa starts at nt 1027 and ending at nt 3315 and presents homology with RNA-dependent RNA polymerase (RdRp) sequences available on the GenBank database. Both ORFs feature over 99% identity with other STV isolates previously reported. Phylogenetic analysis performed with complete STV genomes available on GenBank showed that STV-DR isolate establishes relationship with Americans and Asiatic isolates as well. Due to the low genetic variability, the exact phylogenetic relationship of the Dominican Republic isolate with other STV isolates could not be defined (Fig. 5).

**Fig. 6:**
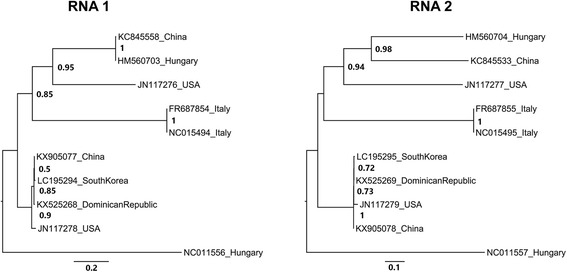
*Pepper cryptic virus 2* (PCV-2) phylogeny. Tree based on complete nucleotide sequences of deltapartitivirus species deposited on GenBank. The isolates are represented in the tree by the accessions numbers of the sequences and the collection point Nodes values with posterior probability > 50% are shown. Sequences were used to analyse the phylogenetic relationship of PCV-2 isolated in Dominican Republic (GenBank accession number KX525268 [RNA1] and KX525269 [RNA2]). Both RNAs of Dominican Republic PCV-2 isolate (GenBank accession number KX525268 [RNA1] and KX525269 [RNA2]) are more phylogenetic related to an American isolate and Asian isolates from China (GenBank accession number KX905078) and South Korea (GenBank accession number LC195295)

A *Pepper cryptic virus 2* (PCV-2-DR) isolate was identified in sweet pepper (*Capsicum annuum* cv. Magali). The PCV-2 belongs to the genus *Deltapartitivirus* (family *Partitiviridae*), presenting a bipartite dsRNA genome [27]. The RNA 1 of the PCV-2-DR has 1.586 kb in size (GenBank accession no. KX525268), while the RNA 2 has 1.534 kb (GenBank accession no. KX525269). The PCV-2-DR RNA 1 presents more than 95% identity with other PCV-2 isolates. PCV-2-DR RNA 2 has 98% identity with other PCV-2 isolates. RNA 1 encodes for a RdRp protein of 478 aa, which is at least 96% identity to RdRp from PCV-2 already reported. It starts at nt 86 until nt 1522. In the RNA 2 there is an ORF coding for a putative coat protein of 430 aa, which begins in nt 9 and ends in nt 1301. Phylogenetic showed that PCV-2-DR RNAs clustered with PCV-2 isolates from USA (GenBank accession numbers JN117278 [RNA1] and JN117279 [RNA2]), South Korea (GenBank accession numbers LC195294 [RNA1] and LC195295 [RNA2]) and China (GenBank accession numbers KX905077 [RNA1] and KX905078 [RNA2]) (Fig. 6).

## Discussion

In this paper, we present the detection of viruses in symptomatic plants from important crops in Dominican Republic by deep sequencing. Viruses from four different genera were identified in a RNA pool isolated from tomato, potato, chilli pepper, sweet pepper and long beans samples. RT-PCR reactions confirmed the presence of those viruses in each plant host. A PCV-2 isolate (PCV-2-DR) was identified in sweet pepper, coexisting with the tospovirus TSWV. A STV isolate (STV-DR) was mix-infecting tomato together with TSWV as well. A BPEV isolate (BPEV-DR) was co-infecting chilli pepper together with TCSV.

The endornaviruses do not show significant nucleotide identity between species, which evidences host/virus co-evolution [21, 28]. BPEV has been described infecting *Capsicum* spp. species [7, 10, 21, 23]. BPEV-DR was identified in *Capsicum frutescens* plants showing chlorotic and necrotic spots, mild curling on leafs and warped fruits (Fig. 1). BPEV infections have not been associated with expressive symptoms. Even when it occurs in co-infection with other viruses, no phenotypic changes have been usually observed, apart from those expected from more symptomatic virus, as TSWV, *Potato virus Y* (PVY) and *Pepper mild mottle virus* (PMMoV) [21]. BPEV-DR is more phylogenetically related to North American isolates, which seem to cause soft symptoms as chlorosis and mild crinkling on young leaves [23]. The symptoms observed in chilli peppers (*C. frutescens*) from Dominican Republic do not seem to be associated with one factor only, since there are more than the usual chlorotic rings induced by tospoviruses in the leaves. It might be possible that the symptoms are due to the mixed infection and/or a virus-synergistic effect between BPEV and TCSV.

PCV-2 has been basically detected in cultivars of *Capsicum annuum* in mixed or single infections, being isolated from symptomatic and asymptomatic plants [29, 30]. PCV-2-DR was isolated from symptomatic sweet pepper plants (Fig. 1), which were concomitantly infected with TSWV. PCV-2 has been reported co-infecting plants with *Cuncumber mosaic virus* (CMV), TSWV, PVY and PMMoV. Single infection of PCV-2 has not caused any visible symptoms [21, 29–33]. The symptoms observed in sweet peppers from Dominican Republic seem to be solely induced by tospoviruses (Fig. 1).

STV has been reported infecting different tomatoes cultivars worldwide [25, 26, 34, 35]. This virus shares biological similarities with other viruses. STV has been associated with a specific phenotype in tomato plants, known as the ‘tomato yellow stunt’ disease [25]. As observed in Dominican Republic samples, STV usually co-exist with viruses from other taxonomical groups like *Tobbaco mosaic virus* (TMV), PVY, TSWV, *Pepino mosaic virus* (PepMV), *Tomato leaf curl virus* (TYLCV), CMV and *Tomato chlorosis virus* (ToCV) [25, 26, 34–38]. Besides that, STV dsRNA has been isolated from both symptomatic and asymptomatic plants, which leads to the question if STV is able to cause any symptom in tomatoes [25, 26]. In Dominican Republic samples STV was detected along with TSWV however, it is not clear if both viruses are responsible for the symptoms observed in the field.

*Tomato chlorotic spot virus* (TCSV) was first reported in tomatoes plants from Brazil in the 1980s [39, 40]. Until recently, TCSV occurrence had been restricted to Argentinean counties and few Brazilian states, without compromising agricultural production [41–45]. However, during the last 4 years, severe TCSV infections have been reported in Caribbean countries (Dominican Republic, Costa Rica and Haiti) and in the south and southeast of the USA [4, 5, 17, 46–49]. Beans, chilli peppers and tomatoes were the first crops affected by TCSV in Dominican Republic [4, 5]. The tospovirus phylogenetic analysis revealed that the TCSV-DR clustered with a TCSV isolate previously reported, when the S and L RNAs are considered, and with the only tospovirus reassortant isolate for RNA M-coded proteins (Fig. 5). The reassortant isolate is a hybrid that has the S and L RNA from GRSV and the M RNA from TCSV [19]. Few years later TCSV has been discovered in USA, it was reported in Central America for the first time [4, 5, 17]. This recent TCSV appearance in Dominican Republic may be due to the plant material trade in the Americas, since imported plants, plants cuttings, seeds and other plant propagative material generally pose the highest risk for introducing foreign plant pests and diseases [50]. Besides that, the World Trade Organization (WTO) forbids the use quarantine rules to protect their markets. This rules allows countries and/or continents, to execute their own regulatory policies, with the commitment that there are not protectionism [51]. Among tospoviruses that infect important crops, TCSV is commonly find in ornamental plants as well, which favours it spread, since these ornamentals species have a high potential to transfer pests and diseases through different geographic regions [52]. According to the Observatory of Economic Complexity (OEC) [1], chilli peppers and others *Capsicum* species are one of the main crop product traded by Dominican Republic. As solanaceous plants, which have been important tospoviruses hosts, these crops became an important virus source.

The recent Dominican Republic (DR) scenario represents an important model to study plant virus interaction and evolution in a restricted area, since multiple plant viruses infections seem to be a common feature and not the exception.

## Conclusions

This work provided the first complete genome of a TCSV isolate from symptomatic plants in Dominican Republic, which was obtained by high-throughput sequencing. Coinfections with three viruses from different virus families were also identified, *Bell pepper endornavirus* (BPEV), *Southern tomato virus* (STV) and *Pepper cryptic virus 2* (PCV-2). The NGS and PCR data showed viruses that have not been described before in DR coexisting in the same host with other important plant viruses, recently reported in the country, as the tospoviruses TCSV and TSWV. The nature and the implications of such interactions are still unknown.

## Abbreviations

BeNMV: Bean necrotic spot virus
BPEV: *Bell pepper endornavirus*
BPEV-DR: *Bell pepper endornavirus* isolated from Dominican Republic
CaCV: Capsicum chlorosis virus
CMV: *Cuncumber mosaic virus*
CSNV: Chrysanthemum stem necrosis virus
DR: Dominican Republic
GBNV: *Groundnut bud necrosis virus*
GP: Tospovirus glycoprotein
GR/TC: *Groundnut ringspot virus*/*Tomato chlorotic spot virus* (reassortant)
GRSV: *Groundnut ringspot virus*
INSV: *Impatiens necrotic spot virus*
IYSV: *Iris yellow spot virus*
L RNA: Tospovirus large RNA segment
L_GRSV_: L RNA from *Groundnut ringspot virus*
M RNA: Tospovirus medium RNA segment
M_TCSV_: M RNA from *Tomato chlorotic spot virus*
MYSV: Melon yellow spot virus
N: Tospovirus nucleocapsid protein
NCBI: National Center for Biotechnology Information
NSm: Tospovirus movement protein
NSs: Tospovirus silencing suppression protein
OEC: Observatory of Economic Complexity
ORF: Open reading frame
PCV-2: *Pepper cryptic virus 2*
PCV-2-DR: *Pepper cryptic virus 2* isolated from Dominican Republic
PepMV: *Pepino mosaic virus*
PMMoV: *Pepper mild mottle virus*
PolRSV: *Polygonum ringspot virus*
PVY: *Potato virus Y*
RdRp: RNA-dependent RNA polymerase
RNA 1: *Pepper cryptic virus 2* first RNA segment
RNA 2: *Pepper cryptic virus 2* s RNA segment
S RNA: Tospovirus small RNA segment
S_GRSV_: S RNA from *Groundnut ringspot virus*
STV: *Southern tomato virus*
STV-DR: *Southern tomato virus* isolated from Dominican Republic
TCSV: *Tomato chlorotic spot virus*
TCSV-DR: *Tomato chlorotic spot virus* isolated from Dominican Republic
TMV: *Tobbaco mosaic virus*
TNSaV: Tomato necrotic spot-associated virus
ToCV: *Tomato chlorosis virus*
TSWV: *Tomato spotted wilt virus*
TYLCV: *Tomato leaf curl virus*
TZSV: Tomato zonate spot virus
WSMoV: *Watermelon silver mottle virus*
WTO: World Trade Organization
ZLCV: *Zucchini lethal chlorosis virus*
